# Methods of Protein Extraction from House Crickets (*Acheta domesticus*) for Food Purposes

**DOI:** 10.3390/foods14071164

**Published:** 2025-03-27

**Authors:** Nair Cunha, Vanda Andrade, Antónia Macedo, Paula Ruivo, Gabriela Lima

**Affiliations:** 1 School of Agriculture, Santarem Polytechnic University, Quinta do Galinheiro-S. Pedro, 2001-904 Santarém, Portugal; 120318002@esa.ipsantarem.pt (N.C.); paula.ruivo@esa.ipsantarem.pt (P.R.); maria.lima@esa.ipsantarem.pt (G.L.); 2 Life Quality Research Centre (CIEQV), Santarem Polytechnic University, Complexo Andaluz, Apartado 279, 2001-904 Santarém, Portugal; atmacedo@ipbeja.pt; 3 Research Centre for Natural Resources, Environment and Society (CERNAS), Santarem Polytechnic University, Quinta do Galinheiro-S. Pedro, 2001-904 Santarém, Portugal; 4 Research Institute for Medicines (iMed.ULisboa), Faculty of Pharmacy, Universidade de Lisboa, 1649-003 Lisboa, Portugal; 5 Polytechnic Institute of Beja—Escola Superior Agrária, Rua Pedro Soares, 7800-309 Beja, Portugal; 6 Mediterranean Institute for Agriculture, Environment and Development (MED), Universidade de Évora, Pólo da Mitra, Apartado 94, 7006-554 Évora, Portugal; 7 LEAF—Linking Landscape, Environment, Agriculture and Food, Higher Institute of Agronomy, Universidade de Lisboa, Tapada da Ajuda, 1349-017 Lisboa, Portugal

**Keywords:** edible insects, crickets, protein extraction, sustainability

## Abstract

Global population is projected to reach 9.1 billion by 2050, emphasizing the need for increased food production. Edible insects, such as house crickets (*Acheta domesticus*), emerged as promising due to higher nutritional value and efficient feed conversion rates compared to conventional protein sources. Incorporating insect powders into new food products can improve consumer acceptance but often leads to poor technological food processing functionality and/or undesirable organoleptic characteristics. Protein isolates have proven to be effective in enhancing this functionality and consumer acceptance, but existent protein extraction methods still lack improvements concerning the optimization of protein extraction rates. This study aimed to address this gap by developing and comparing the yield of three different protein extraction methods using sodium hydroxide, ascorbic acid or alcalase from house crickets (*Acheta domesticus*) for food applications. Protein extraction was performed on cricket powder with a mean protein content of 46.35 g/100 g, and the results were evaluated. The enzymatic method shows the highest protein extraction rate at 69.91% with a mean protein content of 60.19 g/100 g, while extraction with NaOH or ascorbic acid resulted in rates of 60.44 and 46.34%, respectively. Further studies on technological food processing functionality and sensorial evaluation of products developed with this protein extract are recommended.

## 1. Introduction

In recent years, global hunger levels have reached an alarming scale, with projections indicating a growing trend affecting more and more people around the world. This urgency demands immediate and serious action from all nations. According to the Global Report on Food Crises (GRFC) in 2022 [1], the situation has escalated to unprecedented levels: approximately 193 million people in 53 countries/territories suffered from severe food insecurity in 2021, requiring urgent assistance [2]. The Food and Agriculture Organization (FAO) projects that the global population will reach 9.1 billion by 2050, which will require twice the current food production to feed the world, necessitating innovative approaches to ensure adequate food production [3,4].

Traditional protein sources like soybeans, fish, and meat face environmental and scalability challenges [5]. FAO reports from 2021 [6] critically emphasize the impact of traditional practices, including biodiversity degradation, ecosystem damage, and significant contributions to climate change through carbon emissions. Boccardo et al. (2023) highlight that animal protein production, a major component of this demand, is known to have the highest environmental impact in current food production systems [7]. As such, they highlight the need to question and reassess the sustainability of current agrifood systems. Moreover, global dietary trends correlate with increasing rates of chronic non-communicable diseases [8].

A predicted demand for protein has sparked a range of concerns, most notably whether its supply can be met by harvesting from traditional sources of protein alone, such as livestock [9]. It becomes clear that innovative approaches are required to sustainably meet the growing protein demands of the human population. The alternative protein industry is poised to play a significant role in addressing this demand, and through ongoing research, innovation, and investment, it has the potential to contribute to a more environmentally friendly and resilient food system [10].

In this context, edible insects, particularly crickets, have emerged as a promising solution to these challenges due to their rich nutritional profile, efficient feed conversion rates, and lower environmental footprints compared to some traditional protein sources [11,12]. A further advantage of insects as a food source is the high percentage of the animal that can be consumed; up to 80% of a cricket is edible for humans, compared to 55% for pigs and chickens and 40% for cattle [13]. The European Union (EU) estimated that by the year 2054, alternative proteins will make up ≤33% of the global protein consumption, of which insects will account for ∼11% [14].

House crickets (*Acheta domesticus*) are expected to play an important role in the future food systems presenting unique opportunities for improving the food and nutritional insecurity status of both resource-poor and Western populations [15,16]. House crickets are particularly notable for their high protein content (with a higher bioavailability) which ranges from 48.06 to 76.19 g/100 g on a dry basis [12,17]. This makes them an excellent source of protein, comparable and often superior to traditional animal protein sources such as chicken and beef and includes essential amino acids (a.a.) [18,19].

Regarding the environmental benefits of producing crickets for food, various studies have shown that fewer resources are needed compared to traditional protein sources, such as water, feed and space, to produce the same amount of biomass [20,21]. Replacing animal-source foods in current diets with novel foods can reduce all environmental impacts by over 80% and still meet nutrition and feasible consumption constraints [22]. A study performed in southern Australia focused on the carbon footprints of six diverse beef cattle production systems and calculated a range from 10.1 to 12.7 kg CO_2_-eq./Kg^−1^ live weight [23]. In addition, while European farmers are challenged by the need to maintain or increase crop yields and quality facing reduced water availability, agriculture itself is responsible for 70–85% of the global water footprint; as regards livestock production, water footprints of 5988 and 15,415 m^3^/edible ton have already been estimated for pigs and beef cattle, respectively [24,25]. Another study on the environmental potential of insects as a food source in a cricket farm in Finland showed a global warming potential of 7.7 kg CO_2_ eq.g/kg of fresh insects, while a water use of 4341 m^3^/edible ton was estimated from Tapanen (2018) [26]. From a land-use perspective, EntoFarm claims to employ vertical farms, requiring only one m^2^ of land for 100 kg of product, compared to 20,000 m^2^ of land for a comparable amount of beef (EntoFarm, 2023) [27].

Moreover, insects in general have short life cycles, and this makes them highly efficient. For instance, crickets are excellent bio converters that can be fed on low-value organic by-products from the food industry and transformed into high-quality food [20]; in addition, by-products of insect production, including frass, can be high-quality crop amendments [28].

Despite their benefits, the widespread acceptance of edible insects in Western diets remains a challenge due to cultural and psychological barriers and consumer acceptance of insects as a direct food source remains low in Europe [29,30,31]. Western consumers often respond with feelings of disgust and neophobia when presented with the idea of consuming whole insects. In fact, neophobia, or the refusal—in this case—to try new foods, is one of the main factors influencing the acceptability of edible insects. The degree of rejection is related to dislike or disgust, and to a belief that their consumption is associated with cultures from distant and generally low-income countries. The refusal to consume insects is based on cultural reasons, since they are considered unpleasant and, in some cases, harmful, or on doubts about the feasibility and viability of farming them safely. In a study performed in Spain, only 13.15% of participants had tried insects [32]. Another study mentioned that people in Europe remain reluctant to consume insects due to the absence of tradition in this area and the general association of insects with pests, not with food, and documented that in Romania, only 6.3% of participants had already eaten insects [33]. Alhujaili et al. (2023) also mentioned that disgust, visibility of insects, and taste appear to be the most significant factors that can prevent consumers from consuming insects as food. The motivations for acceptance are found to be familiarity and exposure [34].

These barriers have led to a strategic shift in the industry towards incorporating insects into more familiar food forms [20,35]. There is a higher acceptance of insect-based ingredients when they are not visible, such as in the form of powders used in various food products like protein bars, baked goods, and pasta [30]. An EU Consumer Acceptance of Edible Insects: Survey Report, performed in 2024, highlights Europeans’ growing taste for high-protein, vitamin-rich food products that require little resources to produce; it also suggests the need for additional agrifood research [24].

There has been a development of insect powders, particularly those derived from *Acheta domesticus* crickets, due to their potential as a food ingredient and approval for human consumption by the European Commission [36].

Pan et al. [37] describe how the fractionation of components in insect powders, such as protein isolation, can make them significantly more suitable for incorporation into various food products [12].

There are several methods to extract protein from insects, including conventional (e.g., solvents, alkali) and advanced or green extraction methods (e.g., enzyme-assisted extraction) [37]. Protein concentration and isolation from insect powders also include different processes, such as defatting, protein solubilization, or isoelectric precipitation [38]. In fact, lipid removal before insect protein extraction is the most common step reported in the literature, with organic solvents like n-hexane previously applied to different insect species’ lipid removal; petroleum ether is another common solvent currently applied for defatting [39]. However, the neurotoxicity of laboratory solvents such as petroleum ether, benzene and n-hexane is well known [40]. A solvent of interest for defatting is ethanol, which is generally recognized as safe, although the efficiency of ethanol is lower than that of other organic solvents [39]. Alkali extraction is a commonly utilized method in different food matrices, particularly after defatting. Indeed, an alkaline liquid can destroy the secondary bonds of protein molecules, especially the hydrogen bonds, solubilizing the protein molecules and promoting protein separation [41]. As proteins become negatively charged and solubility increases when the pH is raised above the isoelectric point, the insoluble components, which mostly consist of non-protein ingredients, are then removed by centrifugation [42]. Sodium hydroxide (NaOH) is the most used alkali extract. Due to the strong alkalinity and fast ionization rate of NaOH, the NaOH extraction method can usually achieve a higher extraction rate [41]. In consonance, insect proteins are known to be selectively solubilized in an alkaline medium, as the solubility of proteins increases with an increase in pH of the solvent due to the ionization of acidic and neutral a.a. at high pH [37].

The steps for acid extraction are like those for alkaline extraction. With the addition of acid, the pH of the protein solution is gradually lowered below the isoelectric point, a positive charge is generated, and protein solubility increases. Immediately after, the pH is adjusted to the protein isoelectric point and soluble proteins are aggregated and subsequently enriched using precipitation, centrifugation, or filtration [42].

Ascorbic acid may improve the solubility of proteins by disrupting protein–protein interactions that could cause aggregation, making it easier to extract proteins in solution [43]. In fact, reducing agents, such as ascorbic acid, can be used to disrupt, or reduce, disulfide bonds in peptides and proteins [44,45]; protein extraction using ascorbic acid has been previously used for crickets [46].

Although both acids and alkalis are commonly used as a quick and inexpensive method to hydrolyze the degradation of protein peptide bonds, chemicals used during production must be removed before use and may leave residue. Enzymatic hydrolysis can enhance protein extraction yield, increase the degree of hydrolysis, and liberate free a.a., ameliorating the digestibility [47].

The use of enzymatic hydrolysis to obtain bioactive protein hydrolysates has been widely studied, namely using proteases; in fact, the most striking function of proteases is their role in promoting proteolysis [37,48]. Most specifically, alcalase cleaves proteins in the middle of the a.a. chain. Alcalase has been previously used in a wide variety of protein substrates always yielding a high protein hydrolysis degree, either applied individually or in association with other proteases. Even so, a comparison between the efficiencies of alcalase, papain and a commercial cocktail containing trypsin, thymotrypsin, and aminopeptidase in the enzymatic hydrolysis of rice bran protein concentrate and soybean protein showed that alcalase presented a higher capability for hydrolysis, which was about 10 times higher than the other tested enzymes [48]. The use of enzymatic hydrolysis in insect matrices is also described, and the use of alcalase hydrolysis to improve the technological and functional properties of protein flour produced from migrating locusts (*Locusta migratoria* L.) is an example [38,47].

However, the methodologies and conditions selected for each step varied considerably depending on the insect [38]. Research on extracting protein methods has also encountered issues, mainly determining the best method for the insect species to obtain higher yields of protein extraction, which is a challenging issue. Indeed, one significant challenge is the variability in protein yield from different extraction methods. This yield can vary significantly depending on the specific method used, the processing conditions, and the insect species [37]. Concerning crickets, it is namely recognized that many techniques could be applied to enhance the functional properties of cricket protein, such as its foaming properties, solubility, emulsifying properties, water-holding capacity, and oil-holding capacity [49].

Considering all the information above, research on protein extraction methods from such a promising source of protein as *Acheta domesticus*, aiming to enhance the yield of extraction, is still necessary. Therefore, this study aims to develop and select a method to extract protein content from *Acheta domesticus* crickets for food applications by comparing three different methods based on their yields of protein extraction and potential suitability for industrial-scale use. The working hypothesis is that adaptations to previously described methods can increase protein extraction rates.

## 2. Materials and Methods

### 2.1. Chemicals

Ethanol, sodium hydroxide (NaOH), ascorbic acid, hydrochloric acid (HCl) and alcalase (Protease from *Bacillus licheniformis*, ≥2.4 U/g) were purchased from Sigma Aldrich (St. Louis, MO, USA).

### 2.2. Crickets

#### 2.2.1. Crickets Rearing and Harvesting

Whole, frozen, and unpasteurized crickets (*Acheta domesticus*) were obtained from EASYPROTEIN, located in Santarém, Portugal. Crickets were reared in under controlled temperature (approximately 30 °C) and humidity (50–55%). To ensure biosecurity and prevent external contamination, all personnel wore personal protective equipment. Crickets were kept in plastic containers measuring 55 × 39 × 42 cm with population density carefully managed; to promote well-being, egg carton trays were placed inside the boxes [50]. The crickets were fed ad libitum with a commercial poultry feed (PINTOS FEIRA M, Ovopor, Portugal). According to the label, this feed contained approximately 20.6% crude protein, 3.9% crude fat, 1.1% crude fiber, 4.4% total ash, 0.98% calcium, and 0.60% phosphorus.

Harvesting occurred at the end of the cricket’s life cycle (adult stage), with an average of 59 days; adult crickets are fully developed, making them a consistent and standardized raw material for protein extraction. Post-harvest, the crickets were sieved and subsequently euthanized by freezing (−18 °C), a process lasting a minimum of 24 h, and remained there until further processing.

#### 2.2.2. Processing Cricket into Powder

The crickets were processed following established protocols with minor adjustments for optimization. The frozen crickets were defrosted, cleaned to remove extraneous materials, and ground into a paste. This paste was then subjected to drying at 140 °C for 120 min in an oven and further grinding to produce a fine, uniform powder, which was stored under vacuum for further analysis. For each extraction method, 40 g of powder obtained from approximately 160 g of live crickets was weighed.

### 2.3. Study Design

Three distinct protein extraction methods were evaluated and compared for their effectiveness. The first method involved protein extraction using NaOH, following a modified protocol by Zhao et al. (2016) [51]. The second method adopted a protein extraction technique using ascorbic acid described by Amarender et al. (2020) [46]. The third method employed an enzymatic approach to protein extraction using alcalase (protease from *Bacillus licheniformis*) based on the methodology outlined by Hall et al. (2017) [52]. Each extraction method was performed in triplicate to ensure the reliability of the results. For each method, the composition of lipid, carbohydrates, fiber, ash, and moisture content was also assessed (Table 1). The control group was cricket powder without any protein extraction treatment (including the defatting pre-step) considering that the factors under study were the protein content of the cricket powder after protein extraction through the three different methods.

### 2.4. Protein Extraction

Prior to the protein extraction from cricket powder, a lipid extraction process was implemented to enhance protein yield, as described by Gravel and Doyen [53]. This lipid removal step was adapted from the method outlined by Quinteros et al. [54], incorporating specific modifications to optimize efficiency.

The defatting procedure consisted in mixing cricket powder with ethanol in a 1:10 weight/volume (*w*/*v*) ratio, under constant magnetic stirring for 1 h at room temperature, to ensure thorough lipid solubilization. Following this, the ethanol–lipid mixture was separated from the cricket powder, via vacuum filtration. The filtered cricket powder was then dried in an oven at 30 °C for 24 h to achieve complete drying and removal of residual ethanol. The defatted cricket powder, around 30 g for each method, was then used for protein extraction.

#### 2.4.1. Method 1: Protein Extraction with NaOH

A NaOH solution (0.5 M) was mixed with the defatted cricket powder in 50 mL centrifuge tubes at a 6:1 solvent-to-powder ratio (*v*/*w*). The tubes were vortexed and placed in a water bath at 40 °C for 60 min and vortexed every 15 min during the extraction process.

The tubes were then centrifuged at 4 °C for 20 min at 3500× *g* in a Sigma 2K15 centrifuge, and supernatant and any gel layer formed were removed and preserved for subsequent protein recovery steps. A second extraction was performed on the remaining solid insoluble pellet by repeating the vortex and centrifugation steps. The supernatant and gel layer from the second extraction were also saved for protein recovery. The solid pellet was then kept at −80 °C for posterior freeze-drying to reduce the moisture content to less than 5 % (*w*/*w*).

For the recovery of protein potentially lost in the supernatant and gel layer, the collected liquids were adjusted to a pH of 4.3~4.5 using a HCl solution (2 M), which was followed by centrifugation at 4 °C for 15 min at 2500× *g*. The resulting protein precipitate was washed with distilled water and subjected to another centrifugation under the same conditions. In the alkaline medium, proteins are more soluble; hence, the adjustment of the pH allowed for precipitation and recovery of these potentially lost proteins. The obtained pellet was frozen at −20 °C until it could be transported for freeze-drying.

Final extracts were mixed for further analysis and yield calculations.

#### 2.4.2. Method 2: Protein Extraction with Ascorbic Acid

A solution of ascorbic acid (0.5 M) was added to the defatted cricket powder in 50 mL centrifuge tubes at a 6:1 solvent-to-powder ratio (*v*/*w*).

The tubes were vortexed before being placed in a 40 °C water bath for 60 min and vortexed every 15 min to ensure maximum interaction between the defatted cricket powder and the ascorbic acid.

After this step, the tubes were centrifuged at 4 °C for 20 min at 3500× *g* in a Sigma 2K15 centrifuge, and the supernatant and any resultant gel layer were discarded.

An obtained solid insoluble pellet was subjected to a second extraction, which involved a new addition of the ascorbic acid solution (in the same ratio as the first extraction), and vortexing and repeating the centrifugation steps, with the supernatant and gel layer formed during this second extraction also being discarded.

The final insoluble pellet was subsequently frozen at −80 °C and prepared for freeze-drying to achieve a moisture content of less than 5% (*w*/*w*).

Final extracts were mixed for further analysis and yield calculations.

#### 2.4.3. Method 3: Protein Extraction with Enzyme

Alcalase (protease from *Bacillus licheniformis*) was utilized for the enzymatic protein extraction from defatted cricket powder. Distilled water was added to each sample at a 3:1 *v*/*w* ratio, and the mixture was briefly homogenized by vortexing.

The pH of the mixture was adjusted to 8.0, optimal for alcalase activity, using a NaOH (6 M) solution. The samples were heated to 50 °C to facilitate enzyme activity in an orbital stirrer. Alcalase was then added to the mixture at a 3% enzyme-to-substrate ratio (*w*/*w*), and the hydrolysis was conducted for approximately 45 min. To inactivate the enzyme, the hydrolyzed samples were posteriorly heated to 90 °C for 20 min in a water bath.

A centrifugation was conducted at 18,000× *g* for 15 min at 4 °C in a Sigma 2K15 centrifuge and the supernatant was collected, frozen at −80 °C, and prepared for freeze-drying (moisture content of less than 5% dry basis) for further analysis. A second centrifugation round was performed on the remaining solid pellet by adding distilled water at a two-thirds volume relative to the centrifuge tubes. This mixture was vortexed and subjected to a second centrifugation under the same conditions as the first round. The supernatant from this round was also collected, frozen, and freeze-dried.

After freeze-dying, supernatant samples collected from both centrifugation rounds were weighed and pooled for analysis of proximate composition.

For all methods, freeze-drying was performed in a Telstar Lyo quest-85 freeze dryer from Telstar Technologies, S.L. The frozen samples, contained in 50 mL falcon flasks, were placed on the shelves of the freeze-dryer and the vacuum was switched on to allow for a low-pressure environment. Then, heat was applied to convert ice into water vapor, and the temperature and pressure were kept below the triple point of the water (0.01 °C; 0.0060 atm). When most of the ice was removed, the temperature was increased for the removal of unfrozen water molecules. After that, the pressure was reduced to improve drying efficiency. Sample weight was monitored to determine when drying was complete (moist lower than 5%), which was achieved after 3 days.

Calculations of the extraction yield and rate were conducted based on the data obtained from the proximate composition analysis.

#### 2.4.4. Extraction Yield and Rates

The extraction yield is the proportion of material retained after the lipid extraction process relative to the original amount of material. The extraction yield was calculated using Equation (1).

Extraction yield (%) = [(Defatted powder (w)/Initial powder (w)] × 100(1)

where defatted powder refers to the mass of cricket powder remaining after lipid extraction, initial powder is the original mass of cricket powder before any extraction, and protein extraction rate (%) measures the performance of the protein extraction process. It considers the amount of protein obtained from the defatted cricket powder relative to the protein content of the initial material, adjusted by the yield of the extraction process, according to Equation (2).

Protein extraction rate (%) = [Protein content in extract (%)/Protein content in defatted powder (%)] × Extraction yield (%) (2)

where protein content in extract is the percentage of protein in the extract obtained from the defatted cricket powder and protein content in defatted powder is the percentage of protein in the defatted powder before the protein extraction process.

### 2.5. Nutrient Composition

Proximate composition was conducted on cricket powder without any extraction, as well as on the freeze-dried protein extracts obtained from each protein extraction method, for comparison purposes. Nutritional components were analyzed according to the Association of Official Analytical Collaboration (AOAC) [55].

This analysis included the crude protein content (AOAC 950.36) determined using the Kjeldahl method, with a protein-to-nitrogen conversion factor of 5.09; fat content (AOAC 935.38) was measured through Semi-Automatic Soxhlet extraction.; carbohydrates were calculated by difference (100 − [Protein (%) + Fat (%) + Moisture (%) + Ash (%) + Fiber (%)]); and fiber (AOAC 950.37) was analyzed using the Weende method. Finally, moisture content was assessed through gravimetric analysis (AOAC 935.36) and ash content was determined by incinerating the samples to dry ash, also followed by gravimetric measurement (AOAC 930.23).

### 2.6. Statistical Analysis

The results were statistically evaluated using Statistica version 7.0 (StatSoft Inc., Tulsa, OK, United States). An analysis of variance (ANOVA) with one factor (sample) was performed, considering that one-way ANOVA (one factor ANOVA) tests whether there is a difference between the means of more than 2 groups, which were, in this case, NaOH extraction, ascorbic acid extraction, alcalase extraction and the control group [56]. Previously, the Shapiro–Wilk test was applied to check for normality, at a significance level of 5%, to ensure the viability of using parametric tests [57]. For each variable (protein, lipids, carbohydrates, fiber, ash, and moisture), the mean and standard deviation (LS mean) were calculated for each condition and the Fisher LSD post hoc test, which is used as a follow-up to ANOVA [56], was applied to compare means among each one of the groups.

## 3. Results

### 3.1. Nutrient Composition

#### 3.1.1. Cricket Powder (Control)

Among the analyzed nutritional components, protein content (57%) was the highest, followed by fat (19%), fiber (9%), carbohydrates and moisture (6%), and finally ash (4%) (Table 2).

#### 3.1.2. Cricket Protein Extracts

**Figure 1:**
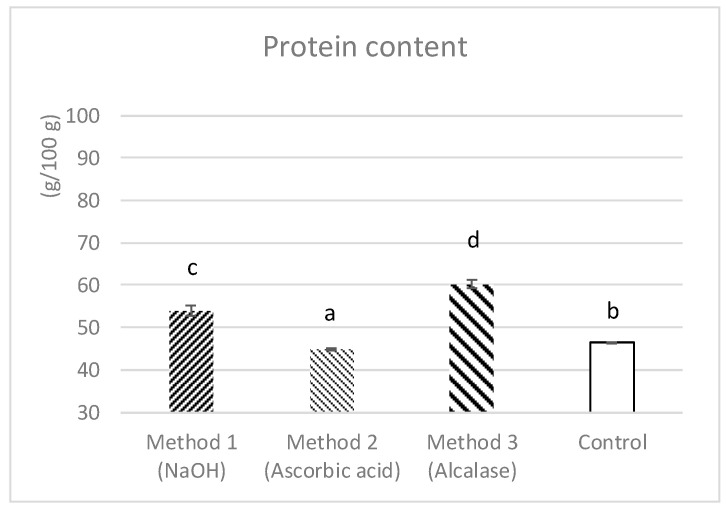
Protein, as g/100 g of cricket powder, in control and after the three extraction methods; sample 1 refers to the chemical extraction with NaOH, sample 2 refers to the chemical extraction with ascorbic acid and sample 3 refers to the enzymatic extraction with alcalase. Data are expressed as (mean ± sd). a, b, c or d is significantly different (*p* < 0.05) from control, samples 1, 2 or 3, respectively.

Related to protein content in the extracts, the enzymatic method (sample 3) showed the highest value which was 60.19 ± 0.95 g/100 g. The alkaline hydrolysis method used for sample 1 yielded better results than the one used for sample 2 (ascorbic acid), with average crude protein contents of 53.93 ± 1.29 g/100 g and 44.92 ± 0.21 g/100 g, respectively; the protein content in sample 2 (ascorbic acid) was lower than that of the initial control sample. The differences among all samples were significant (*p* < 0.05) (Figure 1).

### 3.2. Yields and Protein Extraction Rate

The extraction yields calculated according to Equation (1) were 73.99 ± 4.45%. The protein extraction rates (Equation (2)) varied between 46.30% and 69.91%, with sample 2 having the lowest result as it also had the lowest protein content in the final extract. Sample 3 had the highest protein extraction rate compared to samples 1 and 2. Among the samples obtained, sample 3 showed the best results in yield and protein content of the final extract (Table 3).

### 3.3. Nutritional Composition in Fat, Carbohydrates, Fiber, Ash and Moisture

Protein extraction for all methods (1, 2 or 3) resulted in a significant (*p* < 0.05) lower lipid content than control (19.51 ± 0.1), with values of 5.26 ± 0.2 g/100 g, 0.56 ± 0.03 g/100 g, and 1.66 ± 0.3 g/100 g, respectively (Table 4).

For carbohydrate content, significant (*p* < 0.05) differences were observed among all samples and in the following decreasing order: 26.37 ± 1.39 g/100 g (sample 2), 10.78 ± 0.33 g/100 g (sample 3), 6.75 ± 0.95 g/100 g (control), and undetectable (ND) values (sample 1) (Table 4).

Fiber content in sample 1, obtained from the alkaline hydrolysis, was the highest of all the samples, being 20.32 ± 1.46 g/100 g (*p* < 0.05), followed by sample 2 with 11.51 ± 1.19, extracted with ascorbic acid. In contrast, sample 3, resulting from the enzymatic extraction with alcalase, had the lowest fiber content, 0.70 ± 0.45 g/100 g, which was significantly (*p* < 0.05) lower than the control, which was 9.51 ± 0.57 g/100 g (Table 4).

Regarding ash content, both samples 1 and 3 (extractions with NaOH and with alcalase) showed significantly (*p* < 0.05) higher values than the control: 9.78 ± 0.77 g/100 g, 8.93 ± 0.78 g/100 g and 4.43 ± 0.03 g/100 g, respectively. Sample 2 (extraction with ascorbic acid) had the lowest ash content of all the samples (*p* < 0.05), in the protein extract, at 1.70 ± 0.03 g/100 g (Table 4).

All moisture content values were statistically different from each other (*p* < 0.05). The average values obtained for samples 1, 2, and 3 were 1.26 ± 0.26 g/100 g, 4.55 ± 0.07 g/100 g and 3.9 ± 0.17 g/100 g, respectively, and lower than the value found in the control, which was 6.05 ± 0.09 g/100 g (Table 4).

## 4. Discussion

### 4.1. Nutritional Composition of Cricket Powder

The crude protein content of the cricket powder, obtained in this research, had an average value of 46.00 ± 0.23 g/100 g (Table 2), which is lower than the 65.8 ± 0.52 g/100 g reported by Bassett et al. [59]. This lower value can be justified by the difference in harvesting ages, as the crickets used in this study were harvested within 59 days (around 8 weeks), whereas those used by Bassett et al. [59] were about 5–6 weeks old. However, Ndiritu et al. [60] report that crickets harvested at 10 weeks old, fed with a diet containing 14–21% protein and supplemented with fresh vegetables (green leaves), achieved an intermediate protein content of 59.84 ± 1.64% after processing into powder (freeze-drying and grinding). Besides age, the diet and the nutrients available to the insects throughout their lifecycle can significantly influence their growth rate and nutritional composition. Moreover, under optimal environmental conditions, it is possible to optimize the diet and harvesting age of insects to obtain crickets with higher nutritional value [39,40].

The average fat content was 19.51 ± 0.1 g/100 g (Table 4), similar to Bassett et al. and Ndiritu et al.’s [59,60] 18.1 ± 0.15 and 18 ± 0.07%, respectively, on a dry basis. The lipid content in Acheta domesticus crickets can vary between 8.9 and 43.9% on a dry matter basis [12]. The other components, such as carbohydrates, fiber, and ash, were 6.75 ± 0.95 g/100 g, 9.51 ± 0.57 g/100 g, and 4.43 ± 0.03 g/100 g, respectively (Table 4) and similar values were found for carbohydrates and ash content by Ndiritu et al. [60]—6.39 ± 1.67% and 4 ± 0.06%, respectively; a lower fiber content of 7.16 ± 1.26% was found by the same authors. The carbohydrate content in Acheta domesticus crickets is known to be low, varying between 1.6 and 10.2 g/100 g, similar to the value obtained in the cricket powder in this study. The ash content found in cricket powder agrees with the value reported by Pilco-Romero et al. [12], varying between 1.10 and 5.60 g/100 g on a dry basis, showing the potential of Acheta domesticus crickets to serve as a source of minerals. In addition, Acheta domesticus can also be a source of dietary fiber whose content varies between 3.9 and 7.5 g/100 g (dry basis) [12]. Although this study found a higher value for fiber content, this could be due to the age of the harvested crickets, because it may vary depending on development stage [61]. It is likely that when crickets achieve the adult stage, they do not need to change their exoskeleton (molt) and hardness. The fiber in insects is present in the exoskeleton, where its main component is chitin. It is notable that studies have demonstrated benefits in the human diet resulting from the presence of chitin in insect-derived powders, such as acting as a prebiotic fiber [12,62]. Moreover, insect chitin can be converted into chitosan. According to Ayensu et al. [63], chitosan exhibits high bioactivity and interesting properties such as antimicrobial and antioxidant effects.

### 4.2. Nutritional Composition of Cricket Protein Extracts

One way to improve the acceptance and technological food processing functionality of insect powders is through the fractionation of their different components into extracts, such as protein isolation. The use of the enzymatic method led to the highest protein content, 60.19 ± 0.95 g/100 g, which is lower than the 70.6 ± 0.01 g/100 g found by Hall et al. [52]. However, in the work of Hall et al. [52], total protein content was reported as (% N) using the standard conversion factor 6.25, whereas in our work, the used conversion factor was 5.09 (Table 2) which is recommended for *Acheta domesticus* in a previous study where the chitin, a carbohydrate-containing nitrogen, content was considered [58]. If the conversion value of 6.25 were also used in this study, the obtained value would have been 74.03 ± 0.95 g/100 g, which is higher than the value found by Hall et al. [52]. This indicates that the modifications made to the method described by Hall et al. (2017) [52] during this work were effective in optimizing the parameters to control during extraction. Even so, the method used by Hall et al. [52] was carried out with a different cricket species (*Gryllodes sigillatus*), and the species can be a variation factor in the protein content [19]. Other factors, such as differences in diet or harvesting age, 6 weeks and 8 weeks, respectively, in Hall et al. [52] and in the present study, may have influenced the results obtained. Trinh and Supawong [64] obtained higher protein content in the extract using the same method as Hall et al. [52] and under the same conditions of 85.9 ± 0.7% (*w*/*w*). It is possible that in the latest study, the base material (crickets) had a higher protein content, providing more protein material for the enzyme to act on, during hydrolysis. Protein content in *Acheta domesticus* crickets can vary between 48.06 and 76.19 g/100 g according to the existing literature [12]. This shows that the higher the initial protein content in the raw material, the higher the protein extraction yield (above 90%), which means that the extraction method is feasible for industrial-scale application.

From such a perspective, it is worthy to discuss a plausible reason that can explain why the use of an enzymatic method resulted in a higher protein content than both chemical methods using NaOH or ascorbic acid. Enzymatic hydrolysis is a mild and highly selective biological hydrolysis that does not cause protein denaturation [65]. In this work, alcalase was used, which is an enzyme mainly used to hydrolyze proteins in the food industry, to improve the functional properties of proteins [66]. In fact, protein extraction methods in other matrices such as acid–base treatment are previously mentioned as leading inevitably to protein destruction, which can seriously affect the quality and purity of the extract [65]. However, since the method used to determine protein contents was the Kjeldahl method, which is based on the quantification of the nitrogen content, changes in protein structure would not have any quantitative expression [67]. Rather, we believe that the higher protein content obtained through the enzymatic method might be since alcalase is a proteolytic enzyme that selectively hydrolyzes peptide bonds in proteins, breaking them down into smaller peptides or a.a. [48]. Moreover, several studies reported an improvement in protein solubility by enzymatic hydrolysis [68]. It is plausible that such selectivity combined with increased solubility would improve protein extraction using alcalase when compared with the chemical methods used. Indeed, while alkaline extraction is non-specific and works by altering the pH to solubilize proteins, ascorbic acid (a reducing agent) aids in breaking disulfide bonds in proteins [69]. Both chemical methods lack the targeted action seen in enzymatic hydrolysis, making them less effective and specific.

When comparing the chemical methods used, according to Figure 1, the protein extraction with NaOH provided better results than the use of ascorbic acid, where the average crude protein content was 53.93 ± 1.29 g/100 g and 44.92 ± 0.21 g/100 g, respectively. The protein content of the extracts with NaOH is lower than that of Zhao et al. [51], who obtained 79.0%, on a dry basis. This may be due to the lower concentration of NaOH used in this work and the NaOH solution-to-powder ratio of 0.5 M NaOH and 6:1 (*v*/*w*).

Although alkaline solutions can enhance protein solubility by promoting the dissociation of hydrogen from proteins and the separation of hydrogen bonds, which further improves protein extraction, higher alkali concentrations can, on the contrary, reduce protein extraction [65]. As mentioned, in this work, only one pH media was tested: 0.5 M NaOH and 6:1 (*v*/*w*). Given the low cost of NaOH and the easiness of using NaOH extraction at an industrial scale, further work from our group might include studies on protein extraction with NaOH at different pH.

Regarding the extraction with ascorbic acid, a lower value was obtained compared to Amarender et al. [46], who reported a 69.69% dry basis. The protein content was also lower than that of the initial material (control sample), suggesting that meaningful protein losses occurred during the extraction process, possibly in the supernatant that was discarded. In contrast, the protein lost in the supernatant was recovered when using NaOH, leading to a higher protein content in the final extract. It was expected that the use of ascorbic acid would cause protein precipitation in an acidic medium [70], with the proteins remaining in the solid, insoluble part of the pellet rather than in the supernatant after centrifugation. In this study, protein concentration in the pellet was not observed. It should also be noted that protein precipitation in an alkaline medium resulted in a product with higher protein content compared to the acidic method.

### 4.3. Yields and Extraction Rate

The lipid extraction from cricket powder before protein extraction allowed higher protein extraction rates due to an increase in the initial protein concentration. This provides a greater amount of protein material to be extracted throughout the extraction process, resulting in higher yields.

The extraction yields, or the defatting of the initial mass, varied between 69.10 and 77.81%, with ascorbic acid extraction having the lowest value and alcalase extraction the highest. These values were slightly lower than those found by Amarender et al. and Zhao et al. [46,51]. This can be explained by the modifications made to the lipid extraction method. These lower values may indicate losses in other components, not just lipids. A recent study [71] found lower lipid extraction yields using ethanol compared to hexane, which was more efficient. Additionally, this study also found that ethanol, being an alcohol, could cause protein denaturation and subsequent irreversible aggregation, negatively affecting protein extraction rates. This demonstrates the need for further studies to analyze the structure and profile of protein after the lipid extraction step of cricket powder. Despite the lower yields, ethanol remains a more environmentally friendly and healthier option compared to hexane. Ethanol is produced from renewable resources and has a significantly lower environmental impact, while hexane is derived from non-renewable petroleum sources and poses severe environmental and health risks, including neurotoxicity [72].

The protein extraction rates varied between 46.33 and 69.91%, with the ascorbic acid method having the lowest result as it also had the lowest protein content in the final extract. The enzymatic method had the highest protein extraction rate compared to both chemical methods. This demonstrates the potential for obtaining protein extracts using enzymes, although the method used for sample 1 can also be an alternative as NaOH presents economic advantages as it is cheaper. Similar studies also achieved good results using alcalase to obtain protein extracts with protein contents above 70% dry weight [52,64,73].

While statistical significance confirms that the protein extraction rates differed meaningfully between methods (*p* < 0.05), it is important to consider the practical implications of these variations in a food industry context. The enzymatic extraction method yielded the highest protein recovery (69.91%) compared to the alkaline (60.44%) and acidic (46.34%) methods. Although the ~10% increase in protein yield between the enzymatic and alkaline methods may appear moderate in statistical terms, it represents a substantial improvement in industrial-scale production, potentially increasing protein output per batch while reducing waste and optimizing raw material utilization.

Among the samples obtained, enzymatic extraction showed the best results in yield and protein content of the final extract. Physicochemical and functional property analyses are necessary to validate its use in food applications.

### 4.4. Other Nutritional Components

It was expected that the cricket protein extracts, regardless of the extraction method used, would have a lower lipid content compared to the initial mass (control sample). In accordance, the results showed a significant reduction (*p* < 0.05) in lipid content in all methods, with values of 5.26, 0.56, and 1.66 (g/100 g) for fat content, respectively (Table 4). The results for chemical extraction were much lower than those found by Amarender et al. [46] for fat content, which were 13.34 and 7.62% (*w*/*w*), respectively. As for enzymatic extraction, the lipid content was lower compared to the 4.8 ± 0.1% on a dry basis found by Hall et al. [52].

The results demonstrate the effectiveness of the lipid extraction method prior to protein extraction which involves removing lipids from cricket powder.

For carbohydrate content of all extraction methods and control, significant differences were observed between them (*p* < 0.05). Extraction with NaOH resulted in undetectable (ND) values (Table 4), possibly because NaOH breaks the bonds between proteins and carbohydrates [64]; during centrifugation, the carbohydrates were possibly eliminated in the supernatant. Ascorbic acid extraction leads to the highest carbohydrate values, including control, and the same occurred upon enzymatic extraction (Table 4); this may indicate the presence of proteins bound to carbohydrate molecules, which were transferred to the supernatant (where the protein was concentrated to be isolated).

The mean values found for fiber were statistically different from the control (*p* < 0.05), except for the extraction with ascorbic acid, meaning that in this method, the fiber content was hardly affected (Table 4). Extraction with NaOH resulted in the highest fiber content of all samples, at 20.32 g/100 g dry basis, indicating that NaOH could not act on the fiber components, but instead, there was a concentration of this content in the final extract.

Samples after extraction with alcalase had the lowest fiber content of 0.70 ± 0.45 g/100 g and showed a significant reduction in fiber content compared to the control sample of 9.51 ± 0.57 g/100 g. This can be explained by the action of the enzyme in the hydrolysis of proteins. Chitin is a long-chain polymer of N-acetyl glucosamine present in the insect exoskeleton, where the fiber content is located. Hall et al. [52] verified that the chitin material was successfully removed during hydrolysis and subsequent centrifugation to obtain cricket protein hydrolysates when using alcalase.

Extractions with NaOH or alcalase presented similar ash content results (*p* > 0.05), but were significantly (*p* < 0.05) different, compared to the control and ascorbic acid extracts. As their values were 9.78 ± 0.77 g/100 g and 8.93 ± 0.78 g/100 g, respectively, both showed higher concentrations compared to the control 4.43 ± 0.03 g/100 g. Amarender et al. [46] also found a higher concentration of minerals in the protein extract using the NaOH method, similar to Makishi et al. [74]. Such may be attributed to residual NaOH or its reaction products, such as sodium salts, remaining after the extraction process. NaOH can also provoke the leaching of minerals, either through disruption of the bonds between proteins and minerals or through the solubilization of inherent mineral components present in the cricket powder [75]. Upon using the enzymatic method, a higher ash content can also be explained by using an alkaline solution (NaOH) to adjust the pH necessary for optimizing enzyme activity during hydrolysis. This concentration of ash content in the enzymatic protein hydrolysate was also observed by Hall et al. [52], with values ranging between 9.5 and 14.6% on a dry basis.

Ascorbic acid extraction led to the lowest ash content in the protein extract at 1.70 g/100 g. Ascorbic acid is known to have chelating properties, meaning it can bind to metal ions such as iron (Fe^2^⁺) and copper (Cu^2^⁺) [76]. These minerals are widely found in cricket powders [50,76], by forming stable complexes with these metal ions, ascorbic acid prevents them from interacting with proteins and other components in the solution. During the extraction process, ascorbic acid can chelate these metal ions, keeping them in the soluble fraction (supernatant), which was separated after centrifugation steps and thus impacting the ash content found in this sample.

Moisture content values are statistically different from each other (*p* < 0.05). The values obtained were as expected given that the final dehydration step was freeze-drying with the aim of obtaining extracts with a moisture content of less than 5% (*w*/*w*). The average values obtained after NaOH, ascorbic acid or enzymatic extractions were (g/100 g), 1.26 ± 0.26, 4.55 ± 0.07, and 3.9 ± 0.17, respectively. A lower moisture content, in addition to improving preservation and quality of the protein extract, induces an increase in the concentration values of other components [77,78]. The moisture content values found in this study were like those found by Hall et al., Amarender et al. and Pellerin and Doyen [46,52,71].

The variations in ash and fiber content observed in the extracted protein fractions can significantly influence their functional and nutritional applications in food products. A higher ash content, particularly in the alkaline extraction method, suggests increased retention of minerals, which could enhance the extract’s nutritional profile by providing essential micronutrients like calcium, iron, and magnesium. However, elevated ash levels may also impact flavor, solubility, and processing behavior, potentially leading to an undesirable taste or altered pH in food formulations. This consideration is particularly important for applications in beverages or dairy foods, where a more neutral mineral profile is preferred. In contrast, fiber content was significantly reduced in the enzymatic extraction method, likely due to the removal of insoluble chitin components. This reduction enhances the solubility and smooth texture of the extract, making it more suitable for protein-fortified beverages, emulsified systems, and sports nutrition formulations. On the other hand, higher fiber content, as observed in the NaOH-extracted fraction, may contribute to enhanced water-holding capacity and gelling properties, which could be beneficial for meat alternatives, bakery products, and plant-based food formulations requiring structural integrity.

These findings highlight that while the enzymatic method provides a higher-purity protein extract with improved solubility and digestibility, the alkaline and acidic methods may still offer advantages for specific food applications where higher mineral content or fiber retention is desirable. Future research should further explore these functional differences to optimize cricket protein extracts for targeted food industry applications.

To summarize, this work aims to compare three methods of protein extraction, an enzymatic method using alcalase, and two chemical extractions using NaOH or ascorbic acid. For now, potential biases or limitations should be considered as defatting using ethanol might have contributed to lower yields when compared with organic solvents like n-hexane. Even so, it should be highlighted that the choice of ethanol was consciously made for environmental and consumers’ health reasons even considering the possibility of facing decreased protein extraction rates. Further work using different ethanol/powder ratios and times of extraction is also necessary. In addition, different enzyme/substrate ratios were not tested as well as different NaOH or ascorbic acid concentrations. Is it possible that an optimization of such parameters in each method could lead to different results and higher extraction yields? Such optimization should also be performed considering costs and the perspective of a posterior translation of the methods studied to a larger scale, more specifically to an industrial scale.

A posterior step would be testing the physicochemical functional properties of these protein isolates in different food matrices, such as protein-enriched snacks like protein bars, muffins or cookies, protein shakes for athletes, meat substitutes, considering sensory evaluations to evaluate consumers’ acceptance and preferences, and dietary supplements.

### 4.5. Practical Applications of Cricket Protein Extracts in Food Technology

The growing interest in alternative proteins, particularly insect-derived proteins, presents both opportunities and challenges in food product development. The high-protein cricket extracts obtained in this study, particularly those from enzymatic hydrolysis, offer a promising ingredient for a variety of food applications [37,49]. However, beyond protein yield and purity, their functional, sensorial, and consumer perception aspects must be considered for successful market adoption [30,31].

#### 4.5.1. Food Formulation and Functional Properties

The enzymatic hydrolysis method resulted in the highest protein yield (69.91%) and lowest fiber content, suggesting improved solubility, emulsification, and water-holding capacity, which are desirable properties in beverages, protein shakes, dairy alternatives, and sports nutrition products [37,68]. Higher solubility makes enzymatically extracted cricket protein more suitable for smooth-textured formulations, as opposed to protein isolates with higher fiber content, which may impact texture and dispersion [52,64]. In contrast, the alkaline and acidic extractions retained more fiber, which could contribute to higher viscosity, foaming, and gelation properties, making them potentially more suitable for meat alternatives, bakery products, or texturized plant-based foods [38,49].

#### 4.5.2. Sensorial Profile

One of the key barriers to insect-based protein adoption is the flavor profile, as whole crickets or minimally processed insect powders often have a strong umami, earthy, or nutty taste [32,33,34]. The enzymatic hydrolysis method has been reported in other studies to reduce bitterness and off-flavors by breaking down protein structures into shorter peptides and amino acids, which may enhance salty and umami notes [65,69]. This suggests that enzymatically extracted cricket protein could be more palatable and appealing in clean-label, high-protein products, particularly in plant-based meat formulations or protein-enriched snacks. On the other hand, chemical extractions (alkaline or acidic) may introduce residual flavors depending on the extraction conditions and pH adjustments, which may require additional processing steps (e.g., deodorization, masking agents) for broad consumer appeal [46,70].

#### 4.5.3. Consumer Acceptance and Market Implications

Consumer perception of insect-based ingredients remains a major challenge, particularly in Western markets where neophobia and cultural biases are prevalent [29,30,31]. Studies have shown that consumer acceptance is significantly higher when insects are incorporated as invisible ingredients, such as protein powders or hydrolysates, rather than whole insects [32,33]. The enzymatic method, by improving protein solubility and reducing fiber content, aligns well with the demand for invisible protein fortification in products like protein bars, dairy alternatives, and high-protein bakery products [30,35]. Additionally, enzymatically extracted proteins could appeal to health-conscious consumers, as enzymatic hydrolysis may enhance digestibility and bioavailability, particularly for sports nutrition and clinical applications [47,66].

#### 4.5.4. Potential for Industrial Scale-Up

From a production standpoint, the enzymatic extraction method offers a scalable, food-grade approach that aligns with clean-label trends and minimizes the need for harsh chemicals, making it more attractive for commercial food production [10,37]. However, cost considerations remain an important factor. While enzymatic hydrolysis improves protein recovery and functionality, it requires careful enzyme selection and process optimization to balance yield, cost, and sensory outcomes [52,53,54,55,56,57,58,59,60,61,62,63,64]. Future studies should assess the cost-effectiveness of enzymatic vs. chemical extractions at an industrial scale, considering both nutritional benefits and consumer preference [24,73].

## 5. Conclusions

The findings of this study have direct implications for the food industry, particularly for protein ingredient suppliers, functional food manufacturers, and alternative protein companies. The enzymatic hydrolysis method (69.91% protein extraction rate) produced an extract with improved solubility, reduced fiber content, and a potentially better sensory profile, making it particularly suitable for high-protein beverages, dairy alternatives, and functional sports nutrition products. The alkaline extraction method (60.44% extraction rate), while slightly lower in protein yield, remains a scalable alternative, making it a viable option for meat, plant-based protein formulations, and bakery applications, where structural integrity and water-holding capacity are important. The acidic extraction method (46.34% extraction rate) showed the lowest efficiency, suggesting further optimization is needed before large-scale application. From an industrial perspective, enzymatic extraction provides the highest-quality protein ingredient, though process optimization must be considered for large-scale production. Future work should focus on reducing enzyme costs, improving extraction efficiency, and optimizing functional properties for specific food formulations. Additionally, alkaline extraction could be refined to enhance protein yield, assuring processing costs lower, and making it even more attractive for bulk protein production in cost-sensitive markets.

While this study confirms that cricket protein extraction can yield high-quality functional ingredients, significant regulatory and market challenges remain for industry-wide adoption. In many regions, edible insects are still subject to strict food safety regulations, and consumer acceptance is hindered by cultural perceptions and neophobia. Targeted policy interventions, such as harmonized international food safety regulations, clearer labeling guidelines, and government incentives for sustainable protein production, could facilitate the growth of the insect-based protein sector. Additionally, market education and strategic branding are crucial for consumer acceptance. Studies have shown that consumer perception improves when insect-based proteins are incorporated into familiar food formats rather than whole-insect products. This is why obtaining a protein isolate is essential and more adjustable for direct use in food products. Governments and industry stakeholders should collaborate on awareness campaigns, highlighting the sustainability, nutritional benefits, and functional advantages of insect-derived proteins solving food needs in some populations with no or limited resources. Investment in research and development (R&D) is also needed to optimize processing techniques, enhance taste and texture, and expand the range of applications for cricket protein extracts in mainstream food production.

To further drive industry adoption, research should explore cost-effective scaling strategies for enzymatic protein extraction to improve affordability, functional testing of cricket protein extracts in specific food applications (e.g., emulsification, gelling, foaming), long-term consumer acceptance studies to refine marketing strategies for insect-based proteins, and regulatory alignment across global markets to support commercialization and international trade. By addressing these industrial, regulatory, and consumer-related challenges, insect-based proteins, particularly cricket protein extracts, have the potential to become a mainstream, sustainable alternative to conventional protein sources.

The decision to use ethanol instead of n-hexane was made because we wanted to use a more environmentally friendly solvent with a lower level of toxicity.

By addressing these industrial, regulatory, and consumer-related challenges, insect-based proteins, particularly cricket protein extracts, have the potential to become a mainstream, sustainable alternative to conventional protein sources.

## Figures and Tables

**Table 1:** Experimental design for protein extraction methods.

Method of Extraction	Group	Proximate Analysis (Samples Number)						
		Protein	Lipids	Carbohydrates	Fiber	Moisture		Ash
1	Alkaline	Experimental	3	3	3	3	3	3
2	Acidic	Experimental	3	3	3	3	3	3
3	Enzymatic	Experimental	3	3	3	3	3	3
4	Cricket powder	Control *	5	5	5	5	5	5

* The control was the cricket powder, raw sample, without extraction (common to the 3 methods, always a total of 5 samples).

**Table 2:** Distribution of the nutritional components analysis on the cricket powder (control sample). Results are expressed as % *w*/*w* (mean ± sd) (N = 5).

Nutritional Components of Cricket Powder	
Nutrient	(%*w*/*w*), Dry Basis
Protein *	46 ± 0.23
Fat	19 ± 0.1
Carbohydrates	6 ± 0.95
Fiber	9 ± 0.57
Moisture	6 ± 0.03
Ash	4 ± 0.09

* The Kjeldahl method was utilized for total protein content analysis in food using the universal nitrogen-to-protein conversion factor of 6.25. However, the protein content of insects is likely to be overestimated due to their chitin content, since chitin is a modified polysaccharide that contains nitrogen. Calculations were performed according to the recommendation for *Acheta domesticus* using a factor of 5.09 [58].

**Table 3:** Extraction yield (mass lost after lipid extraction from the initial mass), protein content and protein extraction rate of protein, from defatted cricket powder; protein in defatted cricket powder was 66.99 g/100 g.

Method	Extraction Yield (%)	Protein (g/100 g)	Protein Extraction Rate (%)
1 NaOH	75.07	53.93	60.44
2 Ascorbic acid	69.10	44.92	46.34
3 Alcalase	77.81	60.19	69.91

**Table 4:** Fat, carbohydrates, fiber, ash and moisture as g/100 g of cricket powder, in control and after three extraction methods data are expressed as (mean ± sd). For *p* < 0.05, there are significant differences between samples (different letters) for each parameter. If *p* > 0.05, there are no significant differences between samples (same letters) for each parameter.

	Lipids	Carbohydrates	Fibre	Ash	Moisture
Method 1 (NaOH)	5.26 ± 0.2 (c)	Not detectable	20.32 ± 1.46 (c)	9.78 ± 0.77 (c)	1.26 ± 0.26 (a)
Method 2 (Ascorbic acid)	0.56 ±0.2 (a)	26.37 ± 1.39 (c)	11.51 ± 1.19 (b)	8.92 ± 0.78 (a)	4.55 ± 0.007 (c)
Method 3 (Alcalase)	1.66 ± 0.3 (b)	10.78 ± 0.33 (b)	0.70 ± 0.45 (a)	1.70 ± 0.03 (c)	3.90 ± 0.17 (b)
Control	19.51 ± 0.1 (d)	6.75 ± 0.95 (a)	9.51 ± 0.57 (b)	4.43 ± 0.03 (b)	6.05 ± 0.09 (d)

## Data Availability

The original contributions presented in this study are included in the article. Further inquiries can be directed to the corresponding author.
